# Genome Sequence of *Bluetongue virus* Serotype 17 Isolated in Brazil in 2014

**DOI:** 10.1128/genomeA.01161-16

**Published:** 2016-10-27

**Authors:** Ana Carolina Diniz Matos, Júlio César Câmara Rosa, Kyriaki Nomikou, Lorena Lima Barbosa Guimarães, Érica Azevedo Costa, Maria Isabel Maldonado Coelho Guedes, David Driemeier, Zélia Inês Portela Lobato, Peter Paul Clement Mertens

**Affiliations:** a Universidade Federal de Minas Gerais, Belo Horizonte, Minas Gerais, Brazil; b Vector-borne Viral Diseases, Arbovirus Molecular Research Group, The Pirbright Institute, Woking, Surrey, United Kingdom; c Universidade Federal do Rio Grande do Sul, Porto Alegre, Rio Grande do Sul, Brazil; d School of Veterinary Medicine and Science, University of Nottingham, Sutton Bonington Campus, Leicestershire, United Kingdom

## Abstract

The complete genome sequence of *Bluetongue virus* (BTV) serotype 17 strain 17/BRA/2014/73, isolated from a sheep in Brazil in 2014, is reported here. All segments clustered with western topotype strains and indicated reassortment events with other BTV from the Americas. The strain 17/BRA/2014/73 represents a novel reference strain for BTV-17 from South America.

## GENOME ANNOUNCEMENT

*Bluetongue virus* (BTV) is an arbovirus transmitted between its ruminant hosts by biting midges (*Culicoides* spp.), causing severe hemorrhagic disease in sheep and deer (1). BTV contains 10 double-stranded RNA (dsRNA) genome segments that encode seven structural proteins (VP1 to VP7) and five nonstructural proteins (NS1 to NS4 and S10-ORF2) (2–7). Twenty-seven BTV serotypes have been recognized to date (8, 9).

BTV is widespread around the world (between 40°S and 50°N). Phylogenetic analysis showed that BTV strains evolved separately, with regional variants, named topotypes, for each genome segment. In South America, serological surveys indicated that BTV circulation is widespread (10). BTV-4 has been isolated in northern Argentina and southeastern Brazil (11–14), and BTV-12 was isolated and associated with BT outbreaks in southern Brazil (15, 16). In French Guiana, eight serotypes (BTV-1, -2, -6, -10, -12, -13, -17, and -24) were isolated from ruminants with mild clinical signs or even that were asymptomatic (17). Multiple additional BTV serotypes (BTV-3, -14, -18, -19, and -22) were isolated in Brazil associated with deaths of Brocket deer (*Mazama nana*) in 2015 and 2016 (18). Although there are multiple BTV isolates from South America, complete genome sequences are not available for these isolates, and few phylogenetic analyses have been published (11).

In 2014, an outbreak of hemorrhagic disease, with a mortality rate of 28.89%, affected a flock with 45 Texel sheep in Cachoeira do Sul, Rio Grande do Sul, Brazil (30°02′20″S, 52°53′38″W). BTV was isolated from the blood of one sheep after being passaged three times in the KC cell line and then once in BHK-21 cells. The isolate was identified as BTV-17 by reverse transcription-PCR (RT-PCR) (8) and designated 17/BRA/2014/73.

Genomic dsRNA of the isolate was extracted from infected BHK-21 cells as the template for the synthesis of full-length cDNAs by RT-PCR, and then sequenced on a 3730 ABI capillary sequencer using segment-specific primers (19). Seg-1 to Seg-10 of 17/BRA/2014/73 were 3,944, 2,874, 2,768, 1,981, 1,769, 1,638, 1,156, 1,125, 1,049, and 822 bp, encoding VP1 (1,302 amino acids [aa]), VP2 (955 aa), VP3 (901 aa), VP4 (644 aa), NS1 (552 aa), VP5 (526 aa), VP7 (349 aa), NS2 (354 aa), VP6/NS4 (329/77 aa), and NS3/NS3a/S10-ORF2 (229/216/59 aa), respectively.

Phylogenetic analysis of 17/BRA/2014/73 showed 89.2% nucleotide (nt) identity in Seg-2/VP2 with the African reference strain of BTV-17 (RSArrrr/17, accession no. AJ585138), confirming the isolate as the BTV-17 western topotype (8). All of the other genome segments showed greatest similarity to BTV strains from the Americas subgroup of the major western lineage/topotype. Seg-1/VP1, Seg-6/VP5, and Seg-10/NS3/NS3a/S10-ORF2 were closest (95%, 93.7%, and 99.2% nt identity, respectively) to Argentinian BTV-4 strains from 2001/2002 (strain ARG2002/01, and BTV-4-2001, accession numbers JX024952 and JX024954, respectively). Seg-3/VP3, Seg-4/VP4, and Seg-8/NS2 of 17/BRA/2014/73 were closest (98.3%, 98.1%, and 98% nt identity, respectively) to BTV-12 isolate BRA2002/01. Seg-5/NS1, Seg-7/VP7, and Seg-9/VP6-NS4 of 17/BRA/2014/73 were closest (97.6%, 98.7%, and 98.3% nt identity, respectively) to BTV-2 from USA (2003 to 2010; accession numbers KF986502, JQ822254, and KF986513, respectively). These data suggest that 17/BRA/2014/73 has emerged through reassortment of other BTV strains circulating in the Americas. The strain 17/BRA/2014/73 represents a novel reference strain for BTV-17 from South America.

### Accession number(s).

The genome sequences of BTV-17 strain 17/BRA/2014/73 were deposited in GenBank under the accession numbers KX599359 to KX599368.
